# Supplementary material for: Mechanisms of Trichoderma longibrachiatum T6 Fermentation against Valsa mali through Inhibiting Its Growth and Reproduction, Pathogenicity and Gene Expression
Source: J Fungi (Basel). 2022 Jan 25;8(2):113. doi: 10.3390/jof8020113 (PMC8875883; doi:10.3390/jof8020113)
Supplement: Supplementary file 1 [file jof-08-00113-s001.zip › jof-1498139-supplementary.pdf]

Table S1. Primers for RT-qPCR assay.

Gene ID	Gene name	Function	Primer sequence(F/R)
KUI74297	Gvm2	growth, asexual and sexual development, virulence	CAAAGAGCCCCATCACACAA<br>AAGGTTCGTATCGGTAGCGTT
KUI68175	Gvm3		CGACTACCTGCCCAACGAG<br>GAATCCACTTCTTCCGCTCAC
KUI67703	Pectinase 1	Pectinase	AACGGAGGCACGAAGAAGC<br>CGATGGAGAACAGGTGCGTAG
KUI73936	Pectinase 2		GCGTGGTTTGAGGCTTGTG<br>GTGCCATCCTTCGCTGTTG
KX013493	β-glucosidase1	Cellulase	AGGATAGCAGCACCAACG<br>AACAATGTAACGAAAGGGAC
KUI64411	β-glucosidase2		TGGACCTTCACAGATAATTGGG<br>GAAGTCTACCCAAGTTACGCC
KP229422	Xylanase I	Xylanase	GTGGATACTACTTCTCGTGCTAC<br>GGAGGTTGTCCAGCCATA
ALF05564	VmXyl1		GCCCTCACCCTCGGTATTG<br>GCTGCTTTGAACTGCTGGA
KUI67787	VmVeA	Conidiation, virulence and pectinase	TTCGGTGGGCAGCCTATGT<br>AGATGGGCGGCAGAATGGT
KUI66090	VmVelB		GGATGAAACGGGAACACGG<br>CGCATAGGGACTGACTGGAGAT
KUI66682	Superoxide dismutase		AGGAAATGTGAAGGGTGCC<br>GTGGATGTGGTAGAGGAAAGG
KUI65198	Catalase	Metabolic pathway	ATGGCTGTTTCCCTAAGTGG<br>GCTCCAATTCACCTACATACCG
KUI64284	Citrate synthase		TTATGGATTACGCCTCAGCTC<br>GGGTAGGGATTCTTGGTCTTG
KUI74469	Malate synthase		GTGAGGGCTGATAAGTTGAGG<br>GCTGATTTGGTGTTGGCATG
KUI70334	VmPxE1	Pathogenic effector	GCCCTCACCCTCGGTATTG<br>GCTGCTTTGAACTGCTGGA
KC248180	G6PDH	Reference gene	TCAGAACAAGTTCGAGGGCGACAA<br>TGAGGGCAATAGAGGGCTTGTTCA
KC248178	CYP	Reference gene	GCAAGTCCATCTTCGGTGAG<br>TGGGAGCCGTTGGTGTT
